# Supplementary material for: In Vitro and In Vivo Antiviral Activity of Gingerenone A on Influenza A Virus Is Mediated by Targeting Janus Kinase 2
Source: Viruses. 2020 Oct 8;12(10):1141. doi: 10.3390/v12101141 (PMC7650803; doi:10.3390/v12101141)
Supplement: Supplementary file 1 [file viruses-12-01141-s001.pdf]

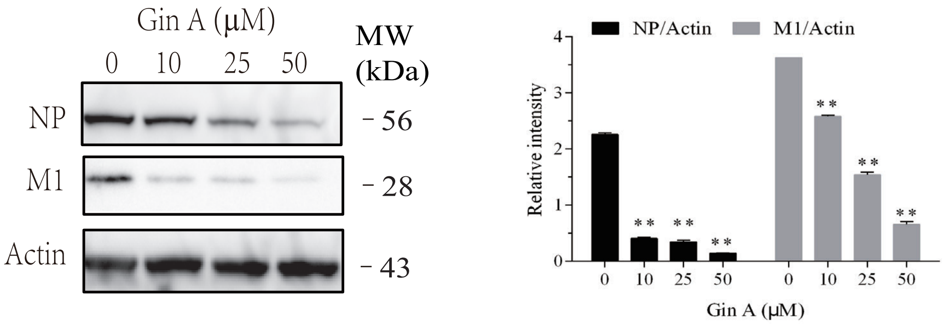

Fig. S1. Gin A inhibits H1N1 viral protein synthesis. 293T cells infected with CA09 strain of the H1N1 virus (0.1 MOI each) were incubated in the absence or presence of the indicated concentrations of Gin A for 12 hr. Cell lysates were prepared and analyzed for NP and M1 expression by Western blot with their specific antibodies. Actin was detected as loading controls. The density of the viral protein bands was analyzed by using NIH Image-J software and normalized by the arbitrary units of Actin. $^{*}p<0.05$, $^{**}p<0.01$, compared to the untreated control.

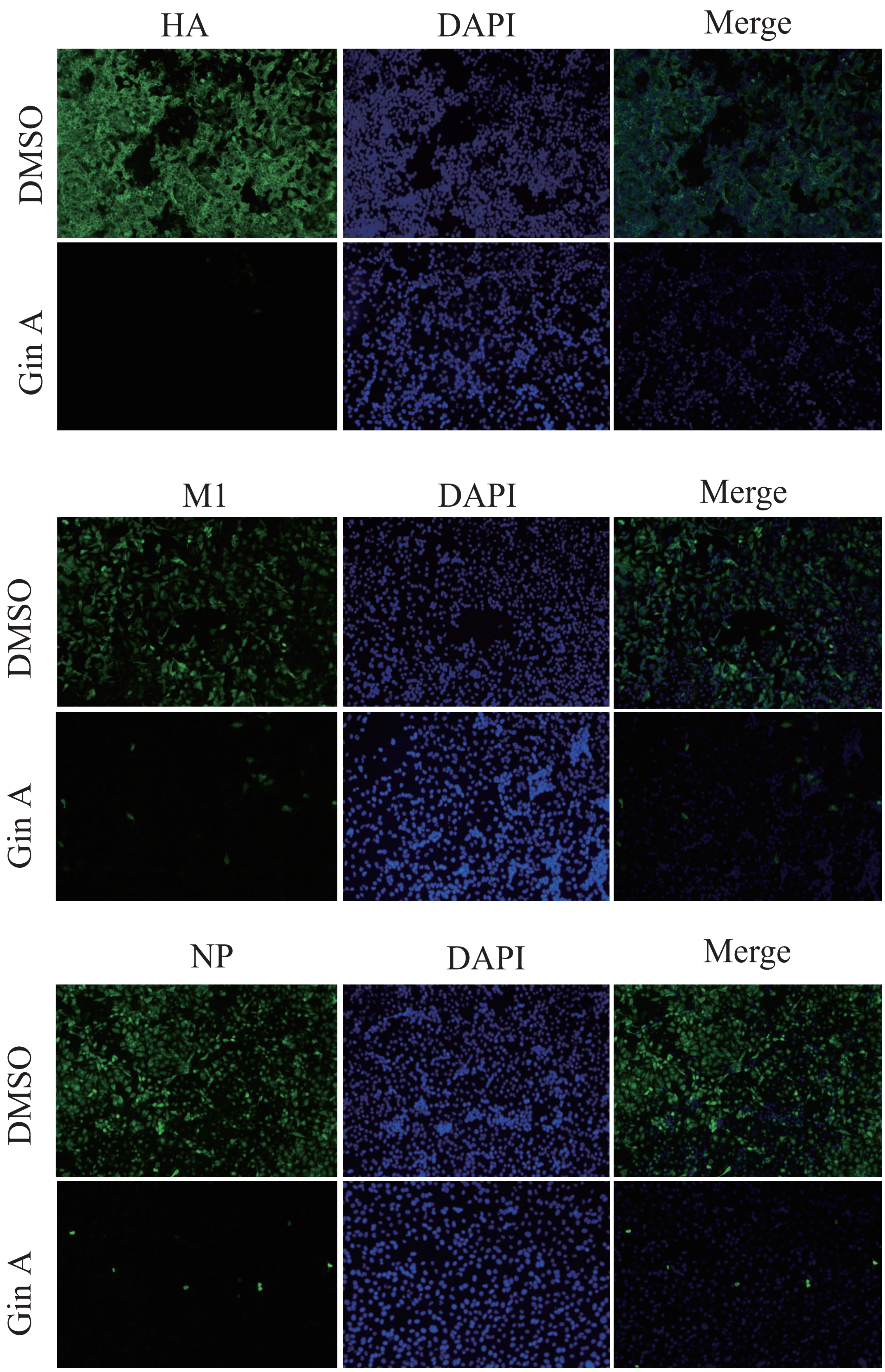

Fig. S2. Gin A decreases the expression of viral proteins. 293T cells infected with H5N1 virus (0.1 MOI) were incubated in the absence or presence of Gin A (25 μM) for 12 hr. Cells were fixed and stained with antibodies against HA, NP, and M1 proteins followed by incubation with Alexa 488-conjugated goat anti-mouse IgG.

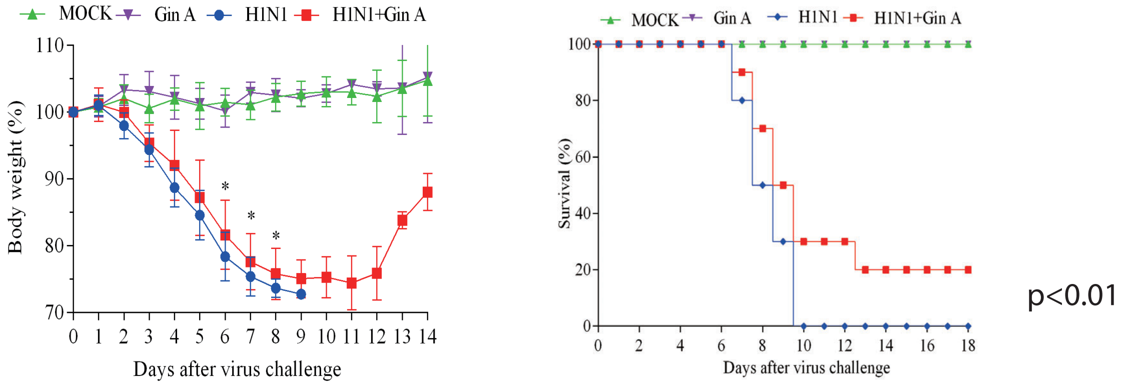

Fig. S3. Gin A inhibits H1N1 virus replication *in vivo*. Female C57BL/6 (6-8-weeks-old) (10 mice per group) were pretreated with Gin A 12 hr by gavage before infection. Mice were intranasally infected with H1N1 virus (100 pfu) and treated with the vehicle (PEG 200) or with Gin A (20 mg/kg body weight) by gavage daily for 7 days. Mice were weighed and monitored for survival for 2 weeks. Body weights (**A**) and percent survival (**B**) were plotted. *$p<0.05$; **$p<0.01$, compared to the untreated controls.
